# A Robust Laser Stripe Extraction Method for Structured-Light Vision Sensing

**DOI:** 10.3390/s20164544

**Published:** 2020-08-13

**Authors:** Congyang Zhao, Jianing Yang, Fuqiang Zhou, Junhua Sun, Xiaosong Li, Wentao Xie

**Affiliations:** Key Laboratory of Precision Opto-mechatronics Technology, Ministry of Education, Beihang University, Beijing 100191, China; zcy1517@buaa.edu.cn (C.Z.); 17374489@buaa.edu.cn (J.Y.); sjh@buaa.edu.cn (J.S.); lixiaosong@buaa.edu.cn (X.L.); fiersies@buaa.edu.cn (W.X.)

**Keywords:** structured-light vision sensor, laser stripe extraction, semantic segmentation

## Abstract

Environmental sensing is a key technology for the development of unmanned cars, drones and robots. Many vision sensors cannot work normally in an environment with insufficient light, and the cost of using multiline LiDAR is relatively high. In this paper, a novel and inexpensive visual navigation sensor based on structured-light vision is proposed for environment sensing. The main research contents of this project include: First, we propose a laser-stripe-detection neural network (LSDNN) that can eliminate the interference of reflective noise and haze noise and realize the highly robust extraction of laser stripes region. Then we use a gray-gravity approach to extract the center of laser stripe and used structured-light model to reconstruct the point clouds of laser center. Then, we design a single-line structured-light sensor, select the optimal parameters for it and build a car–platform for experimental evaluation. This approach was shown to be effective in our experiments and the experimental results show that this method is more accurate and robust in complex environment.

## 1. Introduction

With the development of computer vision and navigation technologies, UGV (unmanned ground vehicle) and MAV (micro aerial vehicle) have come to be widely used in underground inspection, military reconnaissance and device detection [1,2,3]. Global positioning system (GPS) is one of the most popular method for robot navigation tasks. However, for some special circumstances, such as underground mines, under-lit indoors, there is almost no GPS signal due to the enclosed environment. Therefore, it is impossible to use satellite to locate robot. LiDAR scanning allows three-dimensional reconstruction of the surrounding environment but building multiline LIDAR system is way too expensive for the given task.

Considering the above factors, visual sensors are widely used in UGVs and MAVs because of its portability and inexpensiveness. The visual sensors can be categorized into two types: active visual sensors and passive visual sensors. The passive visual sensors are dependent on the ambient light and will fail if the features in the captured image are sparse. As a typical method of active vision, structured-light, due to its low cost, fast acquisition, simple system design, large visual field, has shown great advantages over other methods [4,5,6,7]. Over the course of the past 40 years, many researchers have applied structured-light vision to different tasks. Izquierdo et al. presented a sub-pixel method to measure 3D surfaces based on structured-light projector and calibrated camera [8]. Xie et al. proposed a new approach to calibrate structured-light sensor and apply it to measure the geometric size of certain objects [9]. Liu et al. achieved real time and accurate measurement of rail profile [10]. Fan et al. use line structured light to detect the defect of weld seam [11]. A simple structured-light sensor usually consists of two parts: a camera and a laser projector. The laser projector projects a certain pattern of laser stripes on the objects and the camera will capture the image of the stripe modulated by the front objects. By calibrating the camera initially to get its parameters, we can acquire the objects’ surface information [12,13,14,15]. In structured-light vision inspection, 3D reconstruction and depth measurement can be categorized by the different kind of laser used, such as point laser, line laser and grid laser. This study focuses on the application of single-line structured light.

Locating the laser stripe accurately is a key step for the acquisition of the object depth. However, as the laser beam usually has a certain width of several pixels in the image, we need to extract its center first. Many studies have been conducted for the aim of achieving high precision, applicable efficiency and strong robustness when dealing with complicated environments [16]. These studies can be classified into two following procedures, namely detection and extraction.

**Figure 1:**
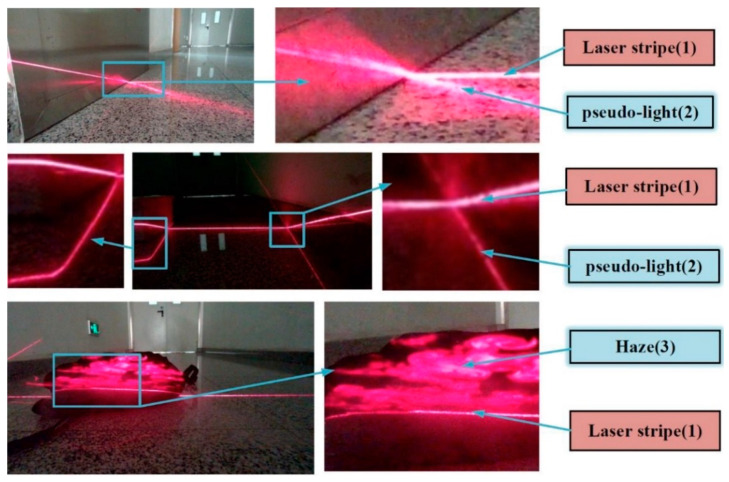
Different laser stripes in real images.

The first step is to detect the location of the laser stripe. To date, none of the methods proposed is perfect and far from being ready to be applied to complicated environments. What caused the noises and bring difficulty to this detection process is that the intensity of the laser stripe that the camera captured is modulated by the interreflections between different surfaces in the environment, the saturation of the laser stripes, some materials like polished metal has extreme reflection capabilities, the incident angles between different surfaces and the uneven surfaces and the discontinuity of line caused by the randomly placed objects in the environment [17]. In other cases, the laser will scatter due to the haze, resulting in the irregular shape of the laser stripe in the image acquired by the camera, as shown in Figure 1.

Some traditional methods detect the region of laser stripe by using RGB color space [18]. However, as the white light also has R component, it is impossible to distinguish the stripe by simply using threshold based on R component. Moreover, there are also some other red pixels due to the interreflection between objects. Hong Nam Ta proposed a novel method [19] to solve the problem of saturation in his study. He takes advantage of YCbCr color space and the laser’s physical properties in order to enhance laser signal and reduce the effects of white ambient light. It also automatically estimates the saturation of laser light and adjusts the exposure by capturing a sequence of images with different exposures.

Some work simply uses the experimental threshold to do the binarization processing, and the result is far from satisfactory. Sun, Q.C. et al. proposed a method using Sobel operator to detect the edge points of laser stripe first [20]. It can only work in ideal environments because Sobel operator cannot distinguish the laser stripe from the noises. Jia Du and Wei Xiong introduced a different approach. They first, propose a ridge segment detector (RSD) which is inspired by LSD to extract the potential laser regions and then rank these regions to find the most possible one [21]. This method is more robust than simply depending on the color information, but still lacks reliability when dealing with the specular reflection area.

Chmelar et al. [22] introduced a novel method of the laser line detection by using well-chosen Gaussian mixture model (GMM). GMM is a method utilizing machine learning. It trains a dataset by giving labels to different pixels. This method is able to solve the problem brought by the different laser intensity in the whole image and reduce saturation’s influence. GMM is based on probability, it ignores the interconnections of pixels and their interior connections, only focusing on the simple information of the pixel itself.

According to the above discussion, these existing methods of extracting the laser stripe center line have some nonnegligible limitations. In recent years, with the rapid development of deep learning, it is common to use deep learning methods to complete advanced visual tasks [23,24]. Krizhevsky et al. [25] proposed AlexNet which is an eight-layer-deep convolutional neural network to solve the problem of image classification, and won the first place in the ILSVRC 2012 competition. AlexNet proved that deep convolutional networks can extract more advanced and effective semantic features in images than traditional methods. Fully convolutional network (FCN) which is a state-of-the-art framework to the semantic segmentation is proposed by Long et al. [26]. Olaf Ronneberger [27] proposed U-net which is an end-to-end semantic segmentation convolutional network in electron microscopic stacks. They won the ISBI cell tracking challenge 2015 in some categories. Kaiming He [28] introduced Mask–R–CNN for instance segmentation. The network first detects the location of the target and then sorts the pixels in the box of target. Vijay Badrinarayanan [29] proposed SegNet which consists of an encoder network and a decoder network. SegNet achieves semantic pixelwise segmentation and encoder network of SegNet extracts rich features. The decoder network’s mission is to map the low-resolution encoder feature maps to full input resolution feature maps for pixelwise classification. Deeplabv3+ is also an encoder–decoder neural network proposed by Liang-Chieh Chen [30]. Deeplabv3+ used ResNet [31] as encoder network to extract features and designed a simple and efficient decoder to restore object boundaries.

Some researchers focus on applying deep learning method to structured-light vision. Li et al. proposed a novel method combining convolution neural network with structured-light measurement [32]. They use deep learning method to achieve stereo matching in occluded environments and can calculate the depth more accurate than traditional methods. Similarly, Du et al. designed SLNet to extract and match features more effectively [33]. This method can also realize real-time depth acquisition. Tao et al. set up a system to measure the box volume based on line structured light and deep learning [34]. They proposed IHED network to extract the edge in the captured image. This method can extract straight line from image efficiently but cannot distinguish laser stripe from other edges.

Though deep learning method has achieved important breakthroughs in semantic segmentation from complex images, few studies have attempted to locate the laser stripe, because there are no big public data set that is adequate to train the deep convolutional neural network well. Moreover, the shape of the laser stripe is relatively slender, and the intersection between the noise region and the laser stripe region is not easy to distinguish. Inspired by DeepLab [30], we propose a novel network to realize highly robust laser stripe region positioning and noise filtering.

The 3D measurement coordinates of real scene are obtained from the image coordinates of the laser stripe’s center according to the measurement model of the structured-light sensor that are described in Section 2. The measurement accuracy of the sensor is highly dependent on the detection accuracy of the light stripe. Moreover, the various interference in complicated environment, such as the pseudo-light and the haze, will severely influence the location and detection of the real laser stripe. Therefore, in practical applications, it is very important and necessary to extract laser stripe center with high robustness and reconstruct 3D point clouds of the stripe position.

In this study, our contributions can be summarized into three aspects:

(1) A laser stripe region segmentation framework based on semantic segmentation network is proposed, which can eliminate the interference of reflective noise and haze noise and realize the highly robust extraction of laser stripes region for the first time;
(2) A dataset representing different noises in sophisticated environments and propose a new strategy for labeling images with laser stripe is set up;
(3) The structured-light vision sensor with single line stripe is designed, selected the optimal parameters for it and built a car-platform for experimental evaluation.

The rest of this paper is organized as follows: Section 2 introduces the measurement model of structured-light sensor. We also design a structured-light sensor, optimize its parameter and finish the calibration process. Section 3 presents the details of our laser-stripe-detection neural network and detection and extraction process in complicated environments. We design and compare different structure of neural network, conduct the performance evaluation test and demonstrate the robustness and availability of our method based on the results of our experiment in Section 4. Section 5 is the conclusion of our work.

## 2. Measurement Model and Design of Structured-Light Sensor

We build a structured-light sensor for robot navigation in the dark and narrow environment at low cost. The hardware part is composed of a monocular camera and a line structured light projector placed next to it and the software part uses the processor to process the raw image to get the point clouds at the position of the light bar, thereby obtaining the information of the environment.

**Figure 2:**
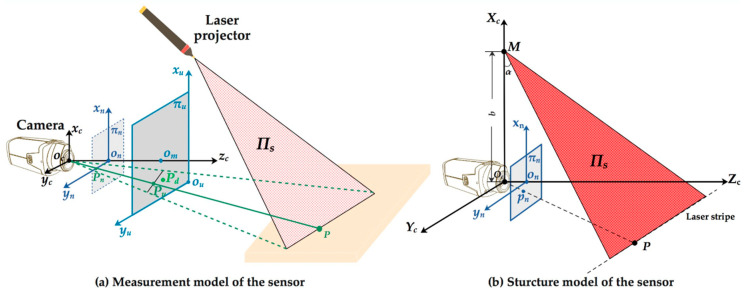
Measurement model and structure model of the structured-light sensor.

The measurement model of the structured-light sensor is shown in Figure 2a. $o_{c}-x_{c}y_{c}z_{c}$ is the 3D camera coordinate system. $o_{n}-x_{n}y_{n}$ is the normalized image coordinate system. $o_{u}-x_{u}y_{u}$ is the undistorted image coordinate system. $\pi_{n}$ is the normalized image plane. $\pi_{u}$ is the undistorted image plane. $\pi_{s}$ is the light plane projected by the laser projector. We set $o_{c}x_{c}//o_{u}x_{u}//o_{n}x_{n}$, $o_{c}y_{c}//o_{u}y_{u}//o_{n}y_{n}$, $o_{c}z_{c}⊥\pi_{u}$ and $\pi_{u}//\pi_{n}$. We assume that P is an arbitrary point in 3D space. The intersection of the ray $o_{c}P$ and the normalized image plane is $P_{n}$, which is the corresponding perspective projection point in $\pi_{n}$. Similarly, $P_{u}$ is the ideal projection point in the undistorted image plane. $P_{d}$ is the real projection point of P in the normalized plane. The deviation between $P_{n}$ and $P_{d}$ is caused by the camera distortion.

We denote the camera coordinate of *P* as $X_{c}={\left[x_{c},y_{c},z_{c}\right]}^{T}$ and its coordinate in normalized camera system as $X_{n}={\left[x_{n},y_{n}\right]}^{T}$. The ideal coordinate of *P* in the image plane is denoted as $X_{u}={\left[x_{u},y_{u}\right]}^{T}$. Then the transformation from $o_{c}-x_{c}y_{c}z_{c}$ to $o_{n}-x_{n}y_{n}$ can be expressed as:

(1) $$X_{n}={\left[x_{c}/z_{c},y_{c}/z_{c}\right]}^{T}$$

We define the focal length in x and y directions are $f_{x}$ and $f_{y}$.respectively. The coordinate of principal point in camera coordinate system is $\left(u_{0},v_{0}\right).$ Then the intrinsic parameter matrix *A* of the camera can be expressed as:

(2) $$A=\left[\begin{array}{ccc} f_{x} & 0 & u_{0}\\ 0 & f_{y} & v_{0}\\ 0 & 0 & 1 \end{array}\right]$$

According to the pinhole model of camera the transformation from $o_{n}-x_{n}y_{n}$ to $o_{u}-x_{u}y_{u}$ can be expressed as:

(3) $$\lambda \tilde{X_{u}}=A\tilde{X_{n}}$$

where λ is the scaling factor and $\tilde{X_{n}}$ and $\tilde{X_{u}}$ are the homogenous coordinate of $X_{n}$ and $X_{u},$ respectively.

The camera we use is not as ideal as the pinhole model. There exist unavoidable distortion and this will diminish the quality of our captured image. In this paper, we take the radial distortion and tangential distortion into account. We consider the first three terms of the radial distortion and the first two terms of the tangential distortion for our model. Moreover, the relationship between $P_{d}$ and $P_{u}\;$ is:

(4) $$\{\begin{array}{c} x_{u}=x_{d}\left[1+k_{1}\left(x_{d}^{2}+y_{d}^{2}\right)+k_{2}{\left(x_{d}^{2}+y_{d}^{2}\right)}^{2}+k_{3}{\left(x_{d}^{2}+y_{d}^{2}\right)}^{4}+p_{1}\left(3x_{d}^{2}+y_{d}^{2}\right)+2p_{2}x_{d}y_{d}\right]\\ y_{u}=y_{d}\left[1+k_{1}\left(x_{d}^{2}+y_{d}^{2}\right)+k_{2}{\left(x_{d}^{2}+y_{d}^{2}\right)}^{2}+k_{3}{\left(x_{d}^{2}+y_{d}^{2}\right)}^{4}+2p_{1}x_{d}y_{d}+p_{2}\left(x_{d}^{2}+3y_{d}^{2}\right)\right] \end{array}$$

where $k_{1},\;k_{2}$ and $k_{3}$ are the coefficients of the lens’ radial distortion and $p_{1},\;p_{2}$ are the coefficients of the lens’ tangential distortion.

In addition, the coordinates of *P* in camera system suit the laser plane’s equation:

(5) $$ax_{c}+by_{c}+cz_{c}+d=0$$

where a,b,c,d represent the coefficients of the laser plane’s equation, respectively.

From the above formula, we can calculate the 3D camera coordinates of the target point independent from the structure parameters of the sensor such as the base distance and tilt angle. Therefore, it can achieve higher accuracy and is more applicable in different environments.

Figure 2b shows the structure design of our sensor. b is the base distance of the sensor. The angle between the laser plane $\pi_{s}$ and the normalized image plane $\pi_{n}$ is α. Moreover, the coordinate systems are same with Figure 2a.

The *z* coordinate of the line where the light plane intersects the ground is the maximum measurement depth $\;Z_{max}$, *x*, *y* are *x* coordinate and *y* coordinate of point $p_{n},$ respectively. We assume that the height from the camera’s optical center to the ground is $h_{c}$ and the pixel error of *x*, *y* $are\;{∆}_{x},\;{∆}_{y.}$ ∆ means the overall error of coordinates of the target point *P*. We take $Z=Z_{max}$, $y=y_{max}$, then we can calculate the target point *P*’s coordinate error, which is shown in Equation (6).

(6) $${∆}_{max}=\frac{Z_{max}}{f}\sqrt{{\left(\frac{h_{c}}{b}+1\right)}^{2}+\frac{\;{\left(y_{max}\cdot \;Z_{max}\right)}^{2}}{f^{2}}+1+\frac{\;Z_{max}}{b^{2}}}\cdot \sigma$$

Through the analysis of the calculation formula of ∆, it can be concluded that the error decreases as the baseline distance increases. According to this conclusion and combined with the actual situation, we finally choose the value of b and optimize the Equation (4) to get the optimal parameters of our sensor.

The result shows that when the baseline distance *b* is 50 mm and the tilt angle α is 70°, our sensor will minimize the coordinate error and not increase greatly in its volume.

The external interface of the sensor is the USB interface of the camera. The sensor is mounted on the car. The image of the light stripe is captured and processed, and the relative position of the UGV and the surrounding environment is obtained, thereby realizing the UGV obstacle avoidance and navigation. The details are in Section 4.

After designing the sensor, we use the dot target to calibrate the sensor and calculate the intrinsic parameter matrix of the camera and the plane equation of the structured-light plane in the camera coordinate system. We also obtain the coefficients of distortion. The quantitative results are shown in Table 1.

## 3. Laser-Stripe-Detection Neural Network and Center Points Localization

### 3.1. Architecture of System and Laser-Stripe-Detection Neural Network

**Figure 3:**
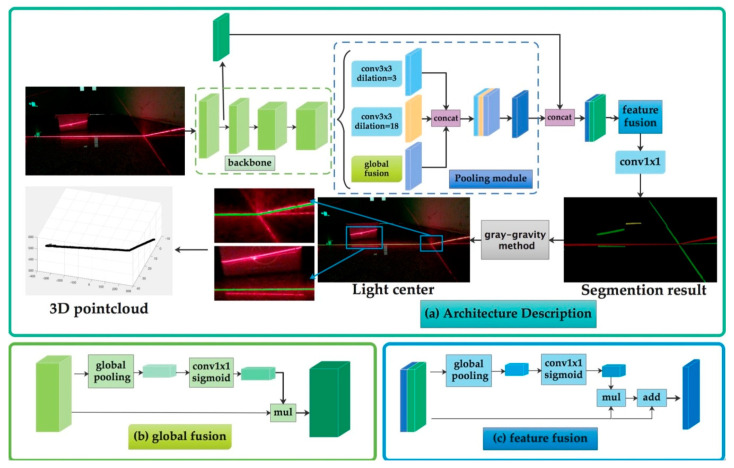
Overall architecture of our system. (**a**) Architecture description of our system. A laser-stripe-detection neural network which is used to detect the resign of laser stripe and denoise and a process which extracts in this part and reconstructs 3D point cloud at the light bar; (**b**) global fusion: a module which uses convolution to merge information with all pixels to enrich detailed information; (**c**) feature fusion: a module which can merge level information and deep level information for better restore space information.

The overall working process of our system is as follows: First, the structured-light projector is used to project the structured light into the environment and the monocular camera is used to capture the image with the light stripe, Second, the region of laser sprite is detected by neural network and then the pixels in the center of the light stripe are extracted by gray-gravity approach from the image which is the output of the neural network. Finally, we use mathematical model in structured-light measurement to reconstruct the point cloud at the light bar to realize the perception of the three-dimensional environment. Figure 3 shows the schematic diagram of our system and Figure 3a shows the process of image segmentation and 3D point cloud reconstruction. The detailed description of the neural network is discussed in Section 3.3.

### 3.2. Image Labeling

Our structured-light sensor projects the line laser into the environment to form a light stripe. Due to the existence of smooth surfaces in the environment, such as marble floor and some metals having extreme surface reflection capabilities, a large number of “pseudo-light stripes” are formed. These “pseudo-light stripes” have similar morphologic features to the real one. Therefore, morphologic modeling cannot be directly applied to extract the stripe. There is also a kind of noise resulting from the scattering of light, usually when there exists haze in the environment. This kind of noise often floods the stripe, causing some obvious morphologic features of the stripe to disappear, making the tradition method fail to detect the accurate region of the laser stripe.

In this paper, the convolutional network is applied to classify the pixels in the image. Each pixel is classified into a certain category. The pixels belonging to the laser stripe area and the pixels belonging to the background area are distinguished. After we finish the segmentation process, the Steger algorithm and the gray-gravity method are, respectively used to further extract the center of the stripe.

**Figure 4:**
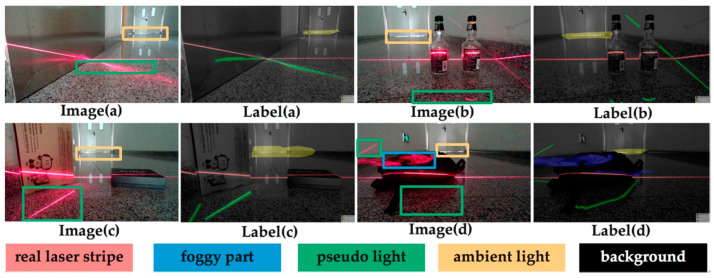
Image labeling.

Since there is a joint between the pseudo-light stripe formed by the reflection and the real light stripe, only labeling the true light stripe cannot successfully achieve the segmentation task. Therefore, the real light stripe and different forms of noise are marked into different categories. Figure 4 shows the schematic diagram of Image Labeling. The real laser stripe part is marked red (first type), the reflective part is marked green (second type), the background is black (third type), the ambient light is yellow (fourth type), and the foggy part is marked blue (fifth type). Images are labeled according to the format of VOC dataset [35].

### 3.3. Structure of Laser-Stripe-Detection Neural Network

Laser-stripe-detection neural network (LSDNN) is a semantic segmentation convolutional neural network which can extract the region of laser stripe. The specific process is as follows: The image captured by the camera (1920 × 1080 pixels) is used as input. First, the ResNet is used to extract rich semantic features as encoder and multiscale dilated convolution as decoder outputs the segmented result.

In order to successfully determine whether a pixel is in the target region or not, a combination of large-scale feature, small-scale feature and global feature is needed. Some traditional methods use multiscale convolution to refine the feature [36]. The accuracy of the network is improved in this way, but the complexity and train time are also increased. Moreover, when the target object has some specific features, such structure may not lead to improvement in network performance.

In this paper, we build a single-line structured-light sensor. Given the fact that the horizontal scale of the laser stripe in image is very large, but its width is relatively small, after extracting feature map by backbone, we only need large-scale convolution and small-scale convolution to extract the features. In order to find the best combination of the number and size of the convolution layers, we conduct an experiment testing different parameters.

**Figure 5:**
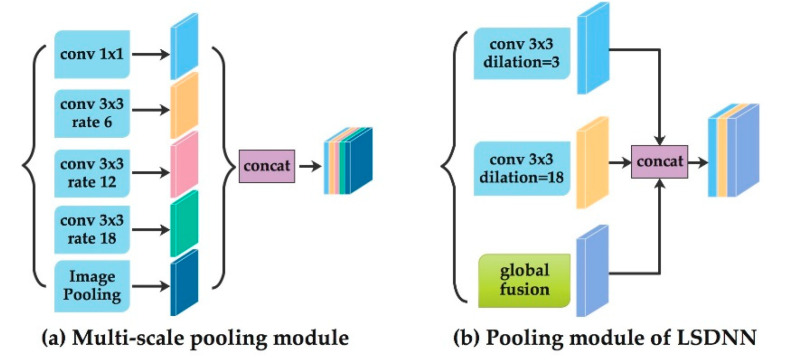
Pooling part. (**a**) Multiscale pooling module; (**b**) pooling module of laser-stripe-detection neural network (LSDNN) that has two dilated convolution layers and one global fusion module.

Figure 5a is a state-of-the-art structure of pooling module in segmentation [30]. It uses multiscale atrous convolution as pooling module to extract higher-level features. We design and compare different structure of the pooling module. The quantitative results are shown in Section 4. The best structure we select for the laser stripe detection is shown in Figure 5b. It has two dilated convolution layers and one global fusion module for pooling. The pooling-module-layer 1 contains a dilated convolution layer which dilation size is 3. It can extract detailed information. The pooling-module-layer 2 contains a dilated convolution layer which dilation size is 18. It can extract large scale information. The global fusion module employs global average pooling to capture global context and computes an attention vector to guide the feature learning. This module can refine the output feature of each stage and provides rich global space information which is useful for laser stripe segmentation.

Then feature-fusion module fuse low-level features and high-level semantic features together. We define the features extracted by ResNet’s first stage as low-level features and the features extracted by multiscale pooling as high-level semantic features. The input of feature-fusion module is the combination of low-level features and high-level semantic features. In this module we balance the scales of the features by the batch normalization and pool the concatenated feature to a feature vector and compute a weight vector. This weight vector can re-weight the features, which amounts to feature selection and combination, and the result we get with this module is much better than the result without it.

Finally, the feature is decoded by upsampling the 3×3 convolutional layer and bilinear difference and a convolution with “1×1 kernel” as decoder layer outputs the segmented result. The detailed architecture of LSDNN is represented in Table 2.

The red region in the segmentation results in the region where the light bar is located, and it is very easy to extract the red region to achieve the extraction of the light bar region in the original image. (Filtering out the interference of reflective noise), the next section will show how to extract the center of the strip from the segment of the stripe.

### 3.4. Training Process

We denote our training dataset as $X=\{x_{i}|i=1,2,\ldots N\}$ and $Y=\{y_{i}|i=1,2,\ldots N\}$. Set *X* is the combination of all laser stripe images in complex environments and set Y is the label image correspondingly. As the LSDNN we propose is an end-to-end network, we use all images in set *X* as the input of our network and the ground-truth image in set Y as the output. This process can be expressed as:

(7) $$X\overset{LSDNN}{\to }Y$$

During the training process, the parameters in our laser-stripe-detection neural network are updated continuously. Each layer has its independent weight parameter and the fusion module fuse them all together. The ultimate goal of our training is to minimize the value of the cost function, which is

(8) $$L=-\overset{N}{\underset{i=1}{\sum }}y^{\left(i\right)}log\left({\hat{y}}^{\left(i\right)}\right)+\left(1-y^{\left(i\right)}\right)log\left(1-{\hat{y}}^{\left(i\right)}\right)$$

here, $y^{\left(i\right)}$ represents the i th ground-truth image and ${\hat{y}}^{\left(i\right)}$ represents the i th prediction image based on $x_{i}$.

### 3.5. Evaluation Method

IoU (intersection over union) is a general evaluation index of semantic segmentation tasks. It represents the ratio of the intersection of two set and their union.

(9) $$IoU=\frac{ground\;truth\wedge prediction}{ground\;truth⊔prediction}$$

When we need to evaluate the accuracy of the task which includes more than one class of object, *mIoU* contains more information because it calculates the mean value of *IoU* over different classes. In our task, as the different categories in the image often have some area of overlaps, we need to focus on the overall segmentation precision instead of just on laser stripe region. *fwIoU* (frequency weight intersection over union) is another indicators which uses the frequency of occurrence of each category as the weight. The mathematical expression of *mIoU* and *fwIoU* are as follow, where *k* is the number of object categories,$\;p_{ij}$ represent the number of pixels whose ground truth are *i*, but predicted result are *j*.

(10) $$mIoU=\frac{1}{k+1}\overset{k}{\underset{i=0}{\sum }}\frac{p_{ii}}{\sum_{j=0}^{k}p_{ij}+\sum_{j=0}^{k}p_{ji}-p_{ii}}$$

(11) $$fwIoU=\frac{1}{\sum_{i=0}^{k}\sum_{j=0}^{k}p_{ij}}\overset{k}{\underset{i=0}{\sum }}\frac{p_{ii}}{\sum_{j=0}^{k}p_{ij}+\sum_{j=0}^{k}p_{ji}-p_{ii}}$$

*mIoU* is regarded as one of the most important indicators in segmentation tasks. Except *mIoU* and *fwIoU* we also use *Acc* (pixel accuracy), *Acc* class (pixel accuracy of class), as the assessment criteria of our experiment. *Acc* represents the correct percentage of pixels and *Acc* class represents the mean value of *Acc* of each category. The mathematical expression of *Acc*, *Acc* class, are as follows:

(12) $$Acc=\frac{\sum_{i=0}^{k}p_{ii}}{\sum_{i=0}^{k}\sum_{j=0}^{k}p_{ij}}$$

(13) $$Acc\;class=\frac{1}{k+1}\overset{k}{\underset{i=0}{\sum }}\frac{p_{ii}}{\sum_{j=0}^{k}p_{ij}}$$

### 3.6. Post Processing Algorithm

The output of the convolutional neural network is a color image of three channels of RGB, wherein the objects of different labels are different in color. When we train the data set, the label of the laser stripe to be tested is designed to be a specific color. Then we only need to traverse all pixels of the output picture and mark the pixel points with specific R channel, G channel and B channel values, the position of the light bar can be accurately extracted. Moreover, the unrelated noise is also filtered out in this way. Because the size and type of the output image are exactly the same as the original image, we can simply filter the stripe area on the basis of traversal and remove other parts to get an image only containing the needed laser stripe.

The intensity distribution of the cross section of the laser tripe usually approximates the gaussian formula [37]:

(14) $$G\left(x\right)=\frac{1}{\sqrt{2\pi \sigma^{2}}}exp[-\frac{{\left(x-\mu \right)}^{2}}{\sigma^{2}}]\;$$

μ is the mathematical expectation and σ is the standard deviation.

For the area to be measured, the normal direction at each place can be obtained by Hessian matrix. The maximum absolute eigenvalue and the corresponding eigenvector of Hessian matrix can be solved to obtain the normal direction of laser stripe and the second derivative in this direction. In addition, Taylor series expansion can be carried out along the normal direction of the stripe since the normal direction is the direction in which the gray scale changes most greatly. Then we can get the center of the stripe by calculating the partial derivative.

Another method for extracting the center line is gray-gravity method (GGM). Similar to the definition of the center of mass in mathematics, each pixel in the image is considered a mass block and the gray value is taken as the mass of each pixel. Each column consists of several pixels can be considered as a “stick”, so the barycentric coordinates of each “stick” is the center line of the laser stripe of this column. Assume the image we get has n rows and m columns. The gray value of the pixel at the i th row and j th column is denoted as I(i,j). Then the center of laser line in the j th column can be expressed as:

(15) $$U_{j}=\frac{\sum_{i=0}i\times I\left(i,j\right)}{\sum_{i=0}I\left(i,j\right)}\;$$

**Figure 6:**
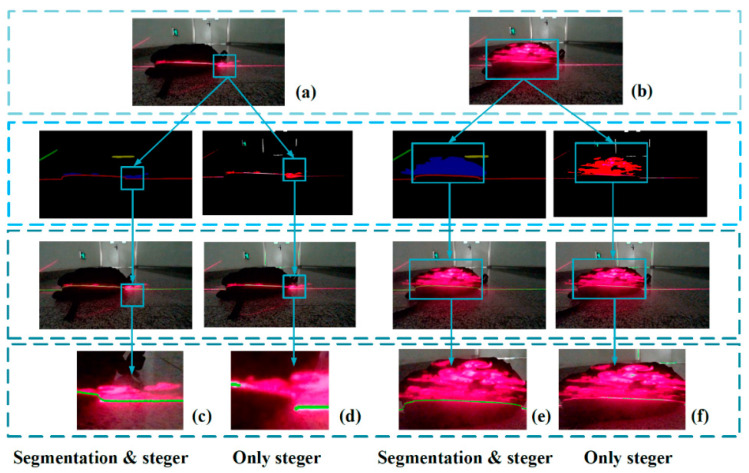
Detection and extraction of laser stripe center. (**a**,**b**) Two different kind of noises. (**c**,**e**) The results of our method(the enlarged region of the center extraction). (**d**,**f**) The results of traditional Steger method.

In this paper, the single-line structured light is used, so there is only one horizontal laser stripe in Figure 6. By using gray-gravity method, the center position of the light stripe in each column can be calculated easily.

Here, we use the above methods to extract the center line of the laser stripe. Steger method is robust, but it is time-consuming. By segmenting the stripe first, we can eliminate the unnecessary time cost as we only need to convolute the selected region of image. The gray-gravity method is fast, but as it takes all pixels into account, it is easily influenced by the noises in image. However, these noises can be filtered from the image by utilizing our method. Figure 6 shows the comparison of our method and Steger method. It can be seen that Steger method fail to detect some part of laser stripe when the haze flooded the target region.

## 4. Experimental Results

**Figure 7:**
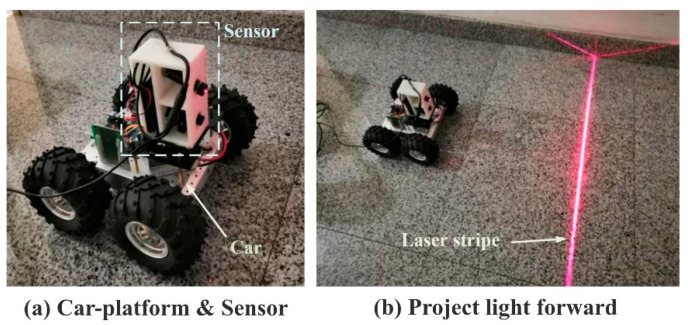
Experiment platform and the experimental scene.

We independently set up a platform for the unmanned car, which is controlled by a single chip microcomputer called Arduino and can be moved remotely by Bluetooth. The structured-light sensor is mounted on the vehicle, and the structured light is projected forward for environmental reconstruction and information perception. The platform is shown in Figure 7.

Experiment in the corridor outside the laboratory and make our own data set for laser strip extraction. Deep learning experiments are conducted using four GTX 2080Ti video cards and other programs are completed under Visual studio 2017.

### 4.1. Discussion and Comparison about Different Structure of LSDNN

We build a dataset independently to train and test LSDNN. There are 5976 images in our dataset in total. We collect and annotate part of the images and the rest images are produced by data augmentation methods. Of the data set, 85% was used for training and 15% for validation.

We use SGD (stochastic gradient descent) as optimizer. ReLU is selected as the activation function in each layer of LSDNN. Table 3 presents the hyper-parameters we used in training process.

LSDNN has two parts, one is backbone for extracting rich semantic features, the other is multiscale dilated convolution as decoder outputs the segmented result. ResNet is one of the best backbones of neural network. We use ResNet which is recognized as a good feature extractor as backbone part of the LSDNN.

The other part of LSDNN consists of multiscale dilated convolution and global fusion module and feature fusion module discussed in Section 3.3. When we classify the different pixels into different categories to successfully detect the laser stripe region, we need to fuse all levels of information together. Multiscale analysis is one of the most powerful tools for extracting different levels of information and augmenting the details of the image. As for our targeted task, the horizontal scale of the laser stripe in image is very large, but its width is relatively small. Therefore, we can combine the small-scale features and large-scale features to achieve higher mIoU. The results of different multiscale convolution layer are shown in Table 4. We can see that the performance of LSDNN is not always better when the convolution module increases. In fact, when we conduct the dilated convolution process, we only need small and large receptive field size. The medium size cannot lead to improvement in mIoU as the specific features of the laser stripe we discussed above. Therefore, we choose the multiscale convolution module with the dilation 3 and 18. Moreover, the global fusion part we design is also essential as it combines the detailed information with overall information.

**Figure 8:**
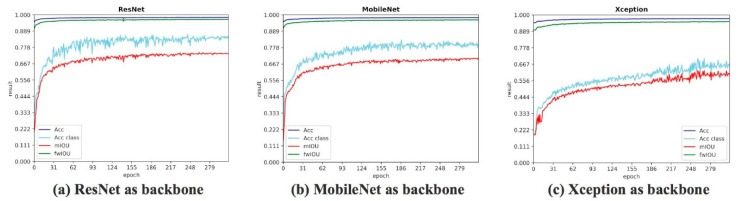
Result of segmentation using different backbone.

In addition to ResNet, Xception and MobileNet were also very useful backbones. We tested these three different backbones. Although mIoU and Acc class fluctuated as the epoch increased, the overall trend of the curve also increased. The results are shown in Figure 8. We found that ResNet performed best among the three backbones. Table 4 is the quantitative comparison result after 300 epochs.

**Figure 9:**
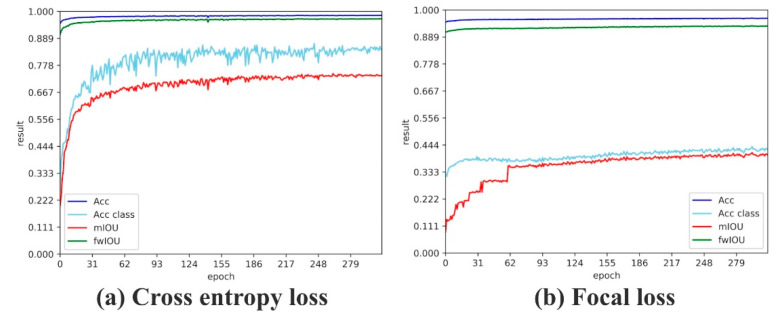
Result of segmentation by different loss function.

Table 5 shows the quantitative results of changing different backbones. According to the above discussion, ResNet worked best. We selected ResNet as the backbone of LSDNN. During the training process, we optimized the loss function to acquire parameters of each layer. We compared two loss functions: cross-entropy loss function and focal loss function. Figure 9 shows the result. We found that cross-entropy loss function achieved a higher value of mIoU, and the overall trend of the curve was smoother. The cross-entropy loss function was more suitable for the target task. Therefore, we selected cross-entropy loss function for further training.

**Figure 10:**
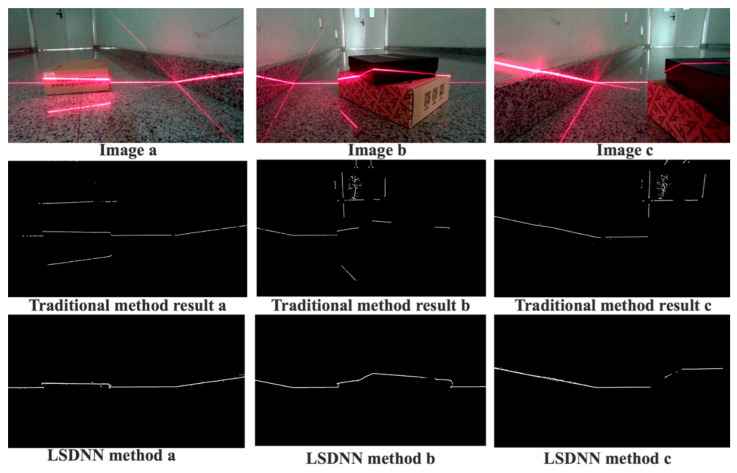
Detection and extraction result of laser stripe center.

After determining the structure of LSDNN, we conducted experiment to further evaluate the performance of our method. Here we used the label image as the ground truth. Then, we calculated the average pixel error of our method and Steger method. The average pixel error was obtained by averaging laser stripe center pixel error in each column. The quantitative results are shown in Table 6. In addition, we compared our laser stripe extraction approach with traditional ones. The “pseudo light”, the noise in environment and discontinuity of line added difficulties to the detection task. Using threshold and morphology method to delete small line and connect some edges was not reliable as it was not adaptive and failed when the image changed. Image a and Image b had a “pseudo light stripe” which could not be easily classified as their shape and intensity were similar. Image c failed to detect and extract some part of laser stripe when the light was cut apart by different objects. In addition to this, noises in the surrounding environment, such as the crack of the door, also had similar properties to light stripe. As shown Figure 10, we found that our method performed better than traditional method and therefore the extraction result could be applied to high-accuracy measurement and navigation tasks.

We ran LSDNN and the postprocessing algorithm on the GPU platform (GTX 2080Ti) and optimized the algorithm to avoid wasting computational resources. Our algorithm could process one image in 82 ms on average. It could meet the needs of robot positioning and navigation.

### 4.2. Detection and Extraction of the Laser Stripe

**Figure 11:**
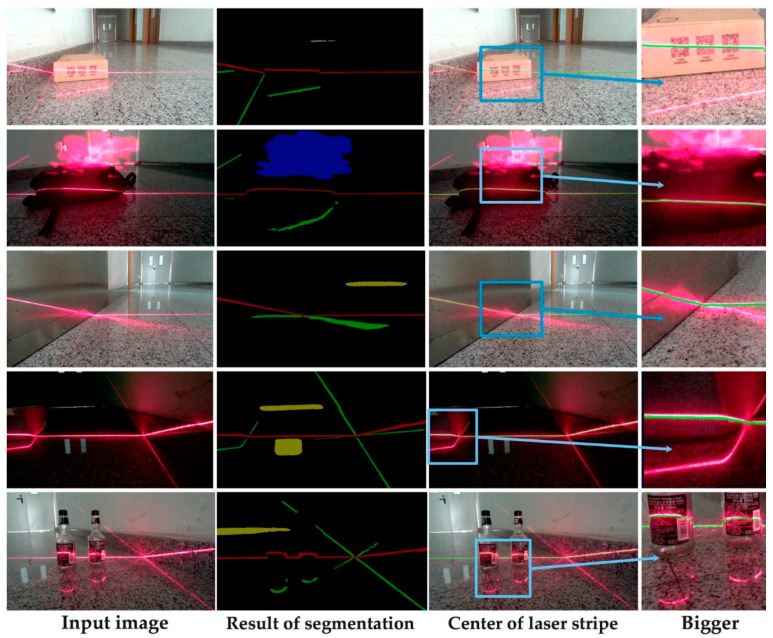
Results of segmentation and extraction dealing with different noises. The input image (1920 × 1920 pixels). The middle column is the output of our network, where different colors denote different categories. The right column is the result of the center extraction based on the segmentation. The laser stripe’s center is marked as green.

Using the network introduced above, we tested many images in different complicated environments. The results are shown in Figure 11. The noises in the image were filtered thoroughly in this way. The “pseudo-stripes” caused by reflection between smooth surfaces were distinguished from the real one. The discontinuity of the laser stripe, the saturation phenomenon and the disturbance resulting from haze were also successfully avoided from influencing the detection and extraction of line center in this way.

### 4.3. Reconstruction of 3D Clouds

**Figure 12:**
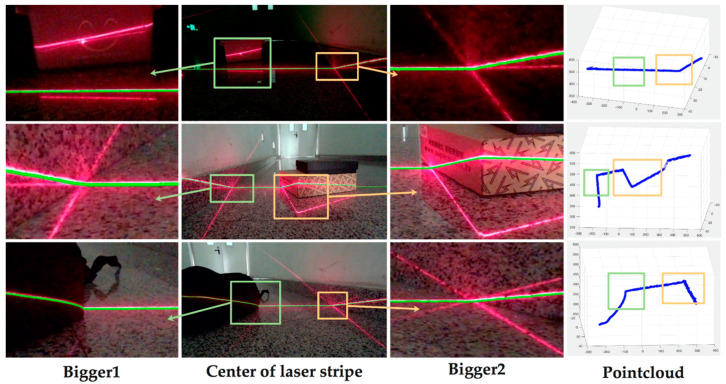
Point clouds reconstructed. The enlarged region in the left is highlighted in the right.

The process to acquire the intrinsic and extrinsic parameters of the camera we used is referred to as calibration [38,39]. The three-dimensional point cloud at the position of the light bar could be obtained by intersecting the ray and the light plane. After the center line of the laser stripe was accurately extracted from the image, we could use the formula mentioned in 4.1 to acquire the three-dimensional coordinates of the center line, which were further used for navigation. The results are shown in Figure 12.

### 4.4. Accuracy Evaluation of the Structured-Light Vision Sensor

**Figure 13:**
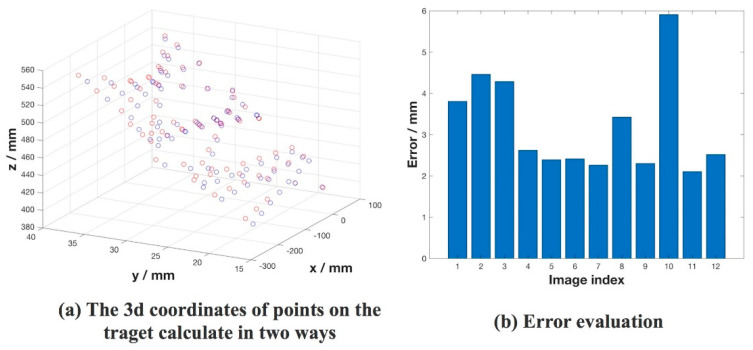
Measurement error evaluation of the sensor.

We set the camera coordinate system as $\;O_{c}-X_{c}Y_{c}Z_{c}$. We selected several points on the intersection of the structured-light plane and the target plane as control points. Then we used the camera’s extrinsic parameters to calculate these points’ three-dimensional coordinates $\left(X_{i}^{c},\;Y_{i}^{c},\;Z_{i}^{c}\right)\;\left(\;i\;\in \left[1,\;7\right]\right)$ in $\;O_{c}-X_{c}Y_{c}Z_{c}$. The results are displayed as the blue dots in Figure 13. Next, we used the measurement model we introduce in Section 2 to calculate the corresponding point’s 3D coordinates $\left(X_{i}^{s},\;Y_{i}^{s},\;Z_{i}^{s}\right)\;i\;\in \left[1,7\right]$ in the camera coordinate system $O_{c}-X_{c}Y_{c}Z_{c}$ which are expressed as the red dots in Figure 13. $\left(X_{i}^{c},\;Y_{i}^{c},\;Z_{i}^{c}\right)\;\left(i\;\in \left[1,\;7\right]\right)$ was closer to the truth value than $\left(X_{i}^{s},\;Y_{i}^{s},\;Z_{i}^{s}\right)\;i\;\in \left[1,\;7\right]$ [40]. Moreover, this paper approximates $\left(X_{i}^{c},\;Y_{i}^{c},\;Z_{i}^{c}\right)\;\left(i\;\in \left[1,\;7\right]\right)$ as truth value. We used the error  E(X,Y,Z) calculated by Equation (16) to evaluate the measurement accuracy of the sensor.

(16) $$E\left(X,Y,Z\right)=\frac{1}{N}\sum_{i=0}^{N}[{\left(X_{i}^{c}-X_{i}^{s}\right)}^{2}+{\left(Y_{i}^{c}-Y_{i}^{s}\right)}^{2}+{\left(Z_{i}^{c}-Z_{i}^{s}\right)}^{2}]$$

The average distance between the measurement point and the calibrated point on the 12 sets of graphs was recorded, which was about 4 mm and this measurement accuracy meets the navigation requirements.

Twelve maps were collected for calibrating the structured-light sensor. Figure 13 shows the measurement error estimations for each image. The measurement accuracy was higher than kinetic and was close to the LiDAR. According to the above discussion and evaluation, using structured light for navigation in the dark environment was a cheap and promising robot navigation method.

## 5. Conclusions

This paper proposes a robust detection method of the laser stripe in complex environment by using deep convolutional network, which is able to deal with different kind of noises. We creatively design the structure of LSDNN and carefully test different structures to achieve the best result. The precision of the extraction is improved significantly, and the time cost is also reduced. We also carry out modeling analysis to design the linear structured-light sensor and use it to realize the environmental sensing of narrow space at low cost and high robustness. In some experimental scenes, the point clouds of the scene can be reconstructed well to obtain the relative position relationship between the robot and the environment. Our future research will be focused on dealing with more diverse noises and optimizing the parameters of our sensor.

## Figures and Tables

**Table 1:** Parameters of the sensor.

Hardware	Parameters	Calibration Result	Physical Meaning
Monocular camera	$\left[\begin{array}{ccc} f_{x} & 0 & u_{0}\\ 0 & f_{y} & v_{0}\\ 0 & 0 & 1 \end{array}\right]$	$\left[\begin{array}{ccc} 1144.5 & 0 & 915.8\\ 0 & 1142.5 & 512.9\\ 0 & 0 & 1 \end{array}\right]$	Camera intrinsic parameters
	[$k_{1}$, $k_{2}$, $k_{3}$, $p_{1}$, $p_{2}$]	[−0.030, 0.00857, 0.00778, −0.000398, 0.0209]	Camera distortion parameters
Line structured light projector	(*a*, *b*, *c*, *d*)	(−0.13, −6.4, 1.0, 303.6)	Laser plane L equation coefficients

**Table 2:** Architecture of LSDNN.

Layer Name	Output Size	Architecture
Input	513 × 513 × 3	/
ResNet-conv1	128 × 128 × 64	7 × 7 convolution
ResNet-conv2	128 × 128 × 64	3 × 3 convolution
ResNet-conv3	64 × 64 × 128	3 × 3 convolution
ResNet-conv4	32 × 32 × 256	3 × 3 convolution
ResNet-conv5	32 × 32 × 512	3 × 3 convolution
pooling-module-layer1	32 × 32 × 256	3 × 3 convolution dilation = 3
pooling-module-layer2	32 × 32 × 256	3 × 3 convolution dilation = 18
global fusion	32 × 32 × 256	global pooling & 1 × 1 convolution
feature fusion	512 × 512 × 5	global pooling & 1 × 1 convolution& sigmoid
output	512 × 512 × 3	/

**Table 3:** Hyper-parameters.

Learning Rate	Batch Size	Optimizer	Activation Function	Loss Function	Epoch
0.007	8	SGD	ReLU	cross entropy	300

**Table 4:** Architecture after backbone.

Conv1×1	Conv3×3	Conv3×3	Conv3×3	Conv3×3	Conv3×3	Global Fusion	*mIoU*
Dilation = 1	Dilation = 1	Dilation = 3	Dilation = 6	Dilation = 12	Dilation = 18		
/	/	/	√	/	√	√	73.86%
/	/	/	/	√	√	√	73.10%
√	/	/	√	/	/	√	74.23%
√	/	√	/	/	/	√	73.29%
/	√	/	/	/	√	√	74.08%
**/**	**/**	**√**	**/**	**/**	**√**	**√**	**74.31%**
√	/	/	/	/	√	√	72.77%
√	/	√	√	√	√	/	72.78%

**Table 5:** Backbone comparison.

Backbone	*mIoU*
ResNet	74.31%
MobileNet	70.04%
Xception	63.14%

**Table 6:** The average pixel error of laser stripe center extraction.

Test Image	Steger Method/Pixels	Our Method/Pixels
Image1	81.8	2.809
Image2	260.9	1.672
Image3	203.6	1.749
Image4	143.6	2.06
Image5	117.8	2.387
Image6	99.45	3.672
